# Supplementary material for: Cooperation of Various Cytoskeletal Components Orchestrates Intercellular Spread of Mitochondria between B-Lymphoma Cells through Tunnelling Nanotubes
Source: Cells. 2024 Mar 30;13(7):607. doi: 10.3390/cells13070607 (PMC11011538; doi:10.3390/cells13070607)
Supplement: Supplementary file 1 [file cells-13-00607-s001.zip › Halasz et al_supplementary_material_revised_changes accepted_2024_03_29.pdf]

Supplementary Material

Cooperation of Various Cytoskeletal Components Orchestrates Intercellular Spread of Mitochondria between B-Lymphoma Cells Through Tunnelling Nanotubes

Henriett Halász[1], Viktória Tárnai[1], János Matkó[2], Miklós Nyitrai[1] and Edina Szabó-Meleg[1,*]

[1]Department of Biophysics, Medical School, University of Pécs, H-7624 Pécs, Hungary

[2]Department of Immunology, Faculty of Science, Eötvös Loránd University, H-1117 Budapest, Hungary

***Correspondence**:

Edina Szabó-Meleg

edina.meleg@aok.pte.hu

Keywords: actin, microtubule, mitochondria, transport, membrane nanotube, motor proteins

Table S1. Primer sequences for siRNA knockdown.

Target genes	Sense strand	Antisense strand
**KIF5B siRNA#3**	5′-GCAAGAAGUAGACCGGAUATT-3′	5′-UAUCCGGUCUACUUCUUGCTG-3′
**KIF5B siRNA#4**	5′-CACGAGCUCACGGUUAUGCTT-3′	5′-GCAUAACCGUGAGCUCGUGTT-3′
**MYO VI siRNA#3**	5′-AGUUCAAGACACAAUUAAATT-3'	5′-UUUAAUUGUGUCUUGAACUTG-3′
**MYO VI siRNA#4**	5′-GCAGGAGAUUGACAUGAAATT-3′	5′-UUUCAUGUCAAUCUCCUGCTG-3′

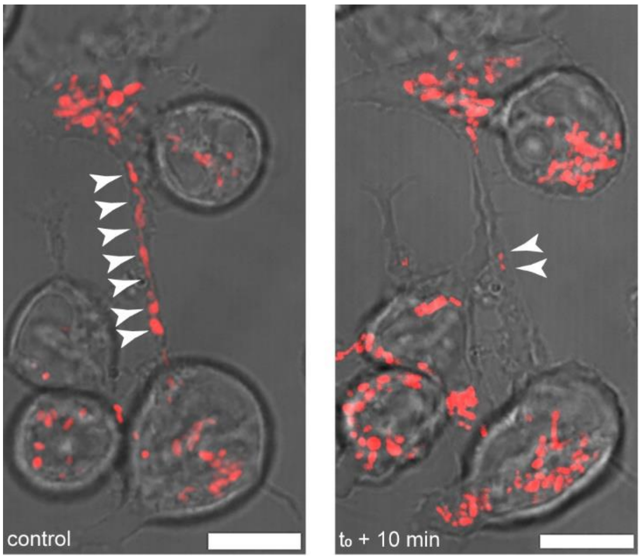

Figure S1. Application of nocodazole in 20 µM concentration disabled the visualisation of mitochondrial transport through B-lymphoma-cell TNTs. The mitochondria escaped from the TNTs within 10 minutes after the addition of the inhibitor. Left: control, right: 10 min-long treatment with 20 µM of nocodazole. Arrowheads depict mitochondria in the examined TNT. Scale bars: 10 µm.

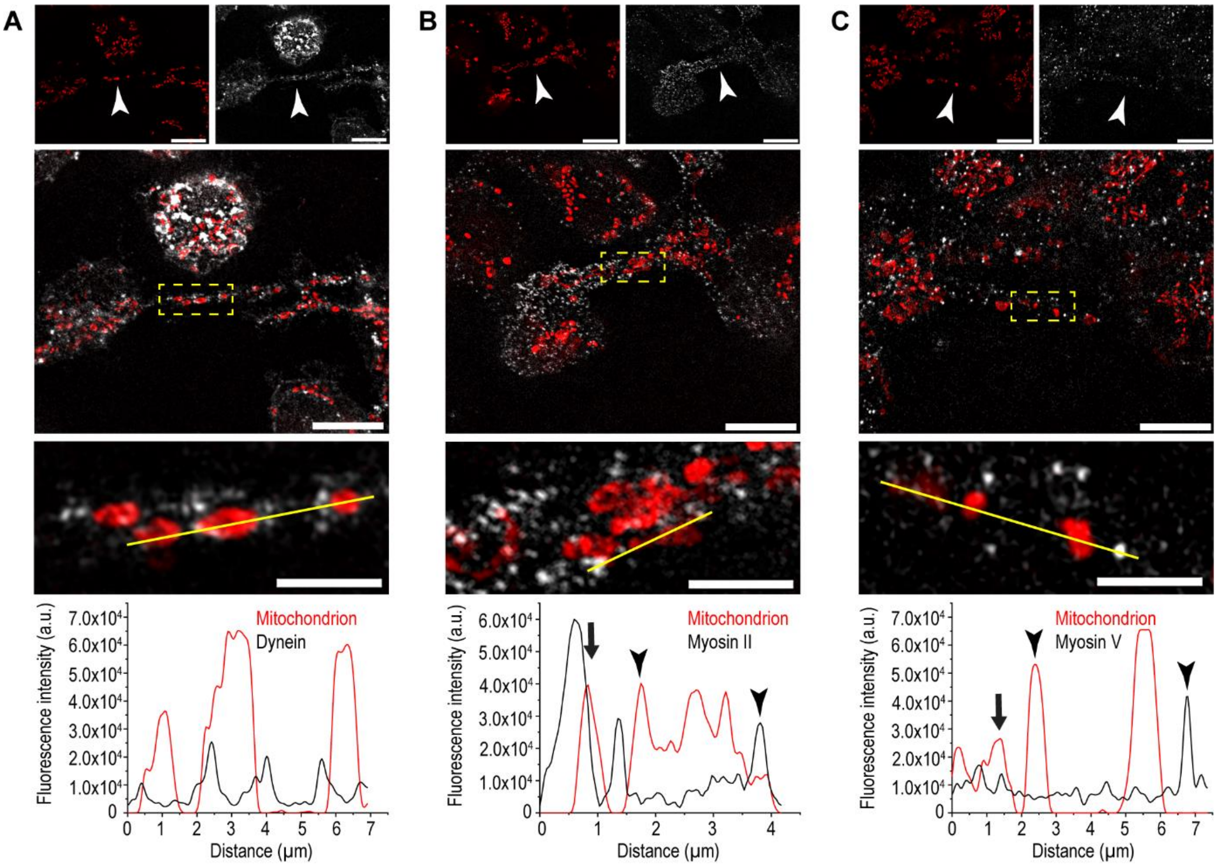

Figure S2. Localisation of mitochondria with dynein, myosin II, and myosin V in B-lymphoma-cell TNTs. (**A**) Representative SR-SIM superresolution images show colocalisation between dynein and mitochondria, which is supported by the line scan analysis; the analysed region is shown by the yellow line. Red: mitochondria, grey: dynein. The micrograph at the bottom is the zooming of the yellow boxed area. (**B**,**C**) Both myosin II (**B**) and V (**C**) sometimes show positional relation with mitochondria. The intensity profiles demonstrate that mitochondria may have a comparable distribution to these myosins (depicted by black arrows), but they can also be located independently of each other (depicted by black arrowheads), the analysed regions are shown by the yellow lines. Red: mitochondria, grey: (**B**) myosin II, (**C**) myosin V. White arrowheads depict TNTs. Micrographs at the bottom are zoomed from the yellow boxed areas. Scale bars: 10 μm and in the zoomings: 3 μm.

Video S1. Mitochondria transport in B-lymphoma-cell TNTs in control conditions. Based on the motion trajectories, the movement of mitochondria is often discontinuous with stopping phases and changes in direction (**A**) rather than straight and continuous (**B**). Red: mitochondria. Trajectories are indicated with blue lines; white spots correspond to tracked mitochondria.

Video S2. Motional trajectories of mitochondrial transport along B-lymphoma-cell TNTs after the inhibition of microtubule polymerisation, the activity of the kinesin and myosin VI motor proteins, or the co-inhibition of the activities of kinesin and myosin VI. Red: mitochondria. The movement pattern is indicated with blue lines, white spots correspond to tracked mitochondria.

Video S3. Movement pattern of mitochondria as a result of dynein, myosin II, or myosin V ATPase activity inhibition. Red: mitochondria. Trajectories are indicated with blue lines, white spots correspond to tracked mitochondria.

Video S4. Motion trajectories of mitochondria due to the depletion of kinesin (KIF5B siRNA#3 and #4) or myosin VI (MYO VI siRNA#3 and #4) expression. Red: mitochondria, green: AF488 siRNAs. The movement pattern is indicated with yellow lines, white spots correspond to tracked mitochondria.

Video S5. Representative time-lapse recordings of A20 cells after the transfection of negative and positive control siRNA constructs. The negative control siRNA did not affect the phenotype of the cells, and the fluorescent signal of the siRNA was visible in TNTs (white arrow). The positive control siRNA silences ubiquitously expressed genes essential for cell survival, consequently inducing a high degree of cell death, as evidenced by blebbing (some blebs are depicted by orange arrows).

Video S6. XZ reconstruction of LifeAct GFP and RFP transfected A20 cells. Optical slicing reveals the presence of partially overlapping actin filaments within B-lymphoma cell TNTs, originating from both contributing cells.
